# Role of vasorin, an anti‐apoptotic, anti‐TGF‐β and hypoxia‐induced glycoprotein in the trabecular meshwork cells and glaucoma

**DOI:** 10.1111/jcmm.17229

**Published:** 2022-02-16

**Authors:** Jin A. Choi, Rupalatha Maddala, Shruthi Karnam, Nikolai P. Skiba, Robin Vann, Pratap Challa, Ponugoti Vasantha Rao

**Affiliations:** ^1^ Department of Ophthalmology Duke University School of Medicine Durham North Carolina USA; ^2^ Department of Ophthalmology College of Medicine St. Vincent’s Hospital The Catholic University of Korea Seoul Korea; ^3^ Department of Pharmacology and Cancer Biology Duke University School of Medicine Durham North Carolina USA; ^4^ Present address: University of California Berkley California USA

**Keywords:** aqueous humour, cell survival, glaucoma, ocular hypertension, trabecular meshwork, vasorin

## Abstract

Glaucoma, one of the leading causes of irreversible blindness, is commonly associated with elevated intraocular pressure due to impaired aqueous humour (AH) drainage through the trabecular meshwork. The aetiological mechanisms contributing to impaired AH outflow, however, are poorly understood. Here, we identified the secreted form of vasorin, a transmembrane glycoprotein, as a common constituent of human AH by mass spectrometry and immunoblotting analysis. ELISA assay revealed a significant but marginal decrease in vasorin levels in the AH of primary open‐angle glaucoma patients compared to non‐glaucoma cataract patients. Human trabecular meshwork (HTM) cells were confirmed to express vasorin, which has been shown to possess anti‐apoptotic and anti‐TGF‐β activities. Treatment of HTM cells with vasorin induced actin stress fibres and focal adhesions and suppressed TGF‐β2‐induced SMAD2/3 activation in HTM cells. Additionally, cobalt chloride‐induced hypoxia stimulated a robust elevation in vasorin expression, and vasorin suppressed TNF‐α‐induced cell death in HTM cells. Taken together, these findings reveal the importance of vasorin in maintenance of cell survival, inhibition of TGF‐β induced biological responses in TM cells, and the decreasing trend in vasorin levels in the AH of glaucoma patients suggests a plausible role for vasorin in the pathobiology of ocular hypertension and glaucoma.

## 1 INTRODUCTION

Glaucoma, an optic neuropathy, is the second leading cause of irreversible blindness worldwide.^1^ Primary open‐angle glaucoma (POAG), the prevalent form of glaucoma, is associated with increased intraocular pressure (IOP)/ocular hypertension, and ocular hypertension is a well‐recognized major risk factor for POAG.^2^, ^3^ Lowering of elevated IOP is a mainstay of treatment for glaucoma.^4^, ^5^ IOP is determined by the balance between aqueous humour (AH) secretion by the ciliary epithelium and AH drainage through the conventional or trabecular pathway consisting of the trabecular meshwork (TM) and Schlemm's canal, and the non‐conventional pathway consisting of the ciliary muscle, supraciliary and suprachoroidal spaces.^6^, ^7^ Impairment in the conventional AH outflow pathway has been recognized to be the main cause for elevated IOP in glaucoma, and experimental elevation of IOP has been demonstrated to induce glaucoma.^6^, ^7^ However, the development of novel efficacious and mechanism‐based IOP lowering therapies is currently hampered by our limited understanding of the aetiological mechanisms involved in ocular hypertension.^5^

Alterations in levels of various external factors including TGF‐β, endothelin‐1, connective tissue growth factor (CTGF), lysophosphatidic acid (LPA), TNF‐α, autotaxin and extracellular matrix in the AH of glaucoma patients have been found to be associated with elevated IOP in glaucoma patients.^6^, ^7^, ^8^, ^9^, ^10^, ^11^, ^12^, ^13^ Several of these external factors have been demonstrated to regulate AH outflow through the trabecular pathway in different experimental models.^6^, ^7^, ^8^, ^9^ Additionally, increased cell death and loss of TM cells in the trabecular pathway have been found to be associated with ocular hypertension in glaucoma.^14^, ^15^ Improvements in proteomics technology have enabled the identification of novel and differentially regulated secreted proteins in AH derived from subjects with different types of glaucoma, indicating the involvement of several external cues in the homeostasis of AH outflow and IOP.^6^, ^16^, ^17^ In this study, the glycoprotein vasorin was identified in almost every sample of non‐glaucomatous human AH (>10 samples tested) we analysed by proteomics analysis. However, we have no knowledge regarding the role and significance of vasorin in AH, and its possible involvement either in the physiology of the TM or in the aetiology of ocular hypertension.

Vasorin, a cell surface single‐pass transmembrane glycoprotein with an estimated molecular mass of ~110 kDa and consisting of 673 amino acid residues, has been shown to be abundantly expressed by vascular smooth muscle cells and moderately by several other tissues and organs.^18^, ^19^ The extracellular portion of vasorin contains several tandemly organized leucine‐rich repeat regions, an epidermal growth factor‐like repeat, a fibronectin type III domain and a short intracellular carboxy terminal peptide with no known sequence homology with other proteins.^18^ Vasorin, a developmentally regulated protein, has been shown to regulate various cellular activities including proliferation, differentiation, migration, angiogenesis, folliculogenesis and calcification and to be involved in the pathobiology of fibrosis, nephropathies and tumorigenesis.^18^, ^19^, ^20^, ^21^, ^22^, ^23^, ^24^ Vasorin has also been demonstrated to bind TGF‐β, and block TGF‐β biological activity^18^, ^25^ and regulate Notch1 signalling by interacting with Numb and preventing degradation of Notch1.^20^ ADAM17 (a disintegrin and metalloprotease 17) has been shown to cleave and release the extracellular portion of vasorin as a soluble and active protein.^25^ Only the soluble/extracellular form of vasorin has been shown to interact with and trap TGF‐β and augment TGF‐β activity under the deficiency of ADAM17.^25^ Moreover, hypoxia‐induced expression of vasorin suppresses TNF‐α mediated apoptosis via regulating mitochondrial thioredoxin activity in mouse embryonic fibroblasts.^21^ While vasorin knockout mice exhibit fertility defects, not much additional knowledge is currently available regarding the physiological role of vasorin.^19^, ^21^

In this study, we determined the levels of vasorin in the AH of POAG patients, expression, secretion and release of the soluble form of vasorin by HTM cells, effects of vasorin on TGF‐β2 mediated cellular events and in TNF‐α induced TM cell death, to gain insights into the role of this glycoprotein in TM biology, AH dynamics and IOP.

## 2 MATERIALS AND METHODS

### 2.1 Human subjects

Research involving the collection of human AH samples has been approved by the Institutional Review Board of Duke University School of Medicine, in compliance with Health Insurance Portability and Accountability Act guidelines and the tenets of the Declaration of Helsinki. Written informed consent was obtained from patients prior to the collection of AH. All samples analysed in the study were obtained from patients who underwent cataract or glaucoma surgeries at the Duke Eye Center.

Patients underwent a review of medical history, measurement of best‐corrected visual acuity and refraction; slit lamp biomicroscopy; gonioscopy and Goldmann applanation tonometer (Haag‐Streit Diagnostics, Essex, UK). In glaucoma patients, disc and red‐free fundus photography, gonioscopy and optical coherence tomography (OCT; Heidelberg Engineering Inc., Franklin, MA) were performed. POAG was defined as the presence of glaucomatous optic neuropathy associated with typical reproducible glaucomatous visual field defects without any other ocular disease or conditions that might elevate IOP, and an open angle on gonioscopy. The exclusion criteria were as follows: a history of any retinal disease; another glaucoma diagnosis, including pigment dispersion syndrome and pseudoexfoliation, and other retinal or optic nerve diseases except for POAG.

### 2.2 Aqueous humour collection

Aqueous humour samples were collected at the initiation of cataract or glaucoma surgery. A tuberculin syringe with a 30‐gauge needle was inserted into the anterior chamber through a limbal paracentesis tract at the start of the surgery, and approximately 30–100 μl of AH was slowly aspirated. The AH samples were transferred from the syringe to a 1.5 ml Eppendorf tube and centrifuged at 1000×g for 10 min at 4°C. The supernatant obtained from the AH samples was collected and stored at −80°C until further use.

### 2.3 Mass spectrometry

Ten µl of AH samples was solubilized in 2% sodium dodecyl sulphate, 100 mM Tris‐HCl (pH 8.0), reduced with 10 mM dithiothreitol, alkylated with 25 mM iodoacetamide and subjected to tryptic hydrolysis using the HILIC beads SP3 protocol (ReSyn Biosciences, Gauteng, South Africa) as described by Hughes et. al.^26^ The resulting peptides were analysed with a nanoAcquity UPLC system (Waters Corp. Milford, MA) coupled to an Orbitrap Q Exactive HF mass spectrometer (Thermo Fisher Scientific, Waltham, MA) employing the liquid chromatography–mass spectrometry (LC‐MS/MS) protocol.

Peptides were identified using Mascot version 2.5.1 (Matrix Science, Boston, MA) for searching a UniProt human revised database (September 2019 release). Mascot search parameters were as follows: 10 ppm mass tolerance for precursor ions; 0.025 Da for fragment‐ion mass tolerance; one missed cleavage by trypsin; fixed modification was carbamidomethylation of cysteine; variable modification was oxidized methionine. Only proteins identified with two or more peptides. Peptide confidence used in this analysis was >80%, which corresponds to a false discovery rate (FDR) <5%. Overall protein confidence was based on the significance threshold of *p*<0.05, which corresponds to a FDR<1%.

### 2.4 Human TM cell culture

Human primary TM cells were derived from TM tissue isolated from donor corneal rings (donors aged 20, 27, 38, 41 and 48 years, with no known ocular complications) used for corneal transplantation at the Duke Ophthalmology Clinical Service, as previously described by us and as per the consensus recommendations for trabecular meshwork cell isolation, characterization and culture.^27^, ^28^ Cells derived from TM tissue were passaged and used in experiments between passages 3 and 6, as we described previously.^27^ TM cells were cultured in Dulbecco's modified Eagle medium containing 10% FBS (fetal bovine serum), PSG (penicillin (100 U/500 ml)–streptomycin (100 µg/500 ml)–glutamine (4 mM)) at 37°C with 5% CO_2_.^27^

### 2.5 RT‐PCR

Total RNA was extracted from passage 4 confluent cultures of TM cells derived from human donors aged 20 and 27 years, using the RNeasy Micro Kit (Qiagen, Valencia, CA, USA). One microgram of total RNA was reverse‐transcribed to prepare single stranded complementary DNA. Expression of vasorin and ADAM17 was determined by RT‐PCR using a C1000 Touch Thermocycler (Bio‐Rad Laboratories, Hercules, CA, USA). The reaction conditions included denaturation at 95°C for 30 s, annealing at 60°C for 30 s and extension at 72°C for 40 s. The cycle was repeated 30 times with a final step at 72°C for five min. The oligonucleotide primer sequences and the expected size of amplified DNA fragments are as below:

**Table:** 

Gene	Forward primer	Reverse primer	Product size (bp)
Vasorin (P1)	CCAACAGGCTGCATGAAATC	AGGCTTAGGTTGCTCACATC	502
Vasorin (P2)	CTGTACTGTGAGAGCCAGATG	GCATGACACAGACGGAGTAA	295
ADAM17 (P1)	CGTGGTGGTGGATGGTAAA	CAAGCTCTTCAGGTGGTTCT	338
ADAM17 (P2)	GTGTCCTACTGCACAGGTAATAG	GGGTGAAACAGAGACAGAGATT	497

The PCR products were sequenced to confirm the identity of amplified DNA.

### 2.6 Enzyme‐linked immunosorbent assay (ELISA)

A human vasorin ELISA kit (MyBioSource, San Diego, CA) was used to determine the levels of vasorin in human AH (duplicates of 10 µl AH were used from each sample), using the manufacturer's protocol which included appropriate standards and background controls.

### 2.7 Immunohistochemistry

To determine the distribution profile of vasorin in the conventional AH outflow pathway, tissue sections derived from formalin‐fixed, paraffin‐embedded human donor eye whole globes (90 year old) were immunostained with a vasorin antibody as we described previously.^29^ Briefly, 5‐μm thick tissue sections were deparaffinized and rehydrated using xylene, absolute ethyl alcohol and water. Antigen epitopes were unmasked by heat‐induced antigen retrieval in 0.1 M citrate buffer pH 6.0 for 20 min at 100°C. The slides were then treated with Biocare Medical's Sniper Background Reducer (Biocare Medical, Concord, CA, USA) to block nonspecific binding. Tissue sections were incubated overnight at 4°C in a humidified chamber with a 1:200 dilution of vasorin antibody (details are described in Table S1). Slides were washed and incubated with Alexa Fluor‐488 goat anti‐mouse secondary antibody (Table S1) for two hrs at room temperature. Immunostained slides were viewed and imaged using a Nikon Eclipse 90i confocal laser‐scanning microscope (Nikon Instruments, Melville, NY, USA). Immunohistochemistry analyses were carried out in duplicates and included a negative control with no primary antibody.

### 2.8 Immunofluorescence

Human TM cells grown on gelatin (2%)‐coated glass coverslips were fixed with 4% paraformaldehyde, permeabilized, blocked and immunostained for vasorin using mouse monoclonal antibody alone or together with TOM20 (Table S1). Additionally, serum starved TM cells (24 h) treated with vasorin (10 ng/ml or 50 ng/ml) for 24 h were stained for F‐actin with phalloidin–Tetramethylrhodamine B isothiocyanate and immunostained for vinculin with a mouse monoclonal anti‐vinculin antibody (Table S1). TM cells treated with CoCl_2_ (0.4 mM) for eight and 24 h were fixed as mentioned above with 4% paraformaldehyde and immunostained for vasorin using an anti‐vasorin antibody and appropriate secondary antibodies conjugated with Alexa fluorophores 488 or 568, as we described previously.^30^ In the above described analyses, samples were incubated with primary antibodies for 24 h at 4°C and with secondary antibodies for two hrs at room temperature. Cell nuclei were counterstained with Hoechst. Finally, coverslips were mounted onto glass slides and imaged using a Nikon Eclipse 90i confocal laser‐scanning microscope.

### 2.9 Immunoblot analysis

Cells were homogenized at 4°C in hypotonic buffer (10 mM Tris buffer, pH 7.4, containing 0.2 mM MgCl2, 5 mM N‐ethylmaleimide, 2.0 mM Na3VO4, 10 mM NaF, 60 µM phenylmethyl sulphonyl fluoride and 0.4 mM iodoacetamide) containing protease and phosphatase inhibitors (one tablet each/10 ml buffer, Roche Pharmaceuticals. Basel, Switzerland), using a probe sonicator and centrifuged at 800×g for 10 min at 4 °C. Samples containing equal amounts of proteins from different treatments, 10 µg for TM cell lysate and CM or in the case of human AH samples, 10 µl from each sample were mixed with 2× Laemmli sample buffer (Bio‐Rad Laboratories, Hercules, California), boiled and separated by sodium dodecyl sulphate–polyacrylamide gel electrophoresis (SDS‐PAGE). Proteins were transferred to nitrocellulose membranes as we described previously.^27^, ^31^ Membranes were blocked for two hrs at room temperature in Tris buffered saline (TBS) containing 5% (wt/vol) nonfat dry milk and 0.1% Tween 20 and subsequently probed overnight at 4°C with respective primary antibodies (see Table S1 for details). Membranes were washed with TBS buffer containing 0.1% Tween‐20 and incubated at room temperature with appropriate secondary antibodies for two hrs. Immunoblots were developed by enhanced chemiluminescence, followed by scanning and analysis using ChemiDoc Touch imaging and Image Lab™ Touch Software (Bio‐Rad Laboratories) respectively. Densitometry analysis was carried out using Image J software.

### 2.10 Cell viability

To determine whether vasorin inhibits TNF‐α induced apoptosis in HTM cells, confluent HTM cultures grown on glass coverslips were treated with 30 ng/ml human TNF‐α (Sigma‐Aldrich, St. Louis, MO), and 1 µg/ml cycloheximide (Sigma‐Aldrich, St. Louis, MO. USA) with or without 50 ng/ml vasorin for 24 hrs as described Choksi et al.^21^ Live cells were then briefly washed with 1X PBS and incubated with propidium iodide (PI), 0.5 µg/ml in serum‐free DMEM media for 5 min at 37°C. PI is a membrane impermeant dye that is generally excluded from viable cells, while intercalating with double‐stranded DNA in cells undergoing apoptosis. Images of red fluorescent positive cells were captured under a fluorescent microscope (10×, Zeiss Axioplan 2). A minimum of 10 images were captured at different locations on the same coverslip, and the number of fluorescent positive cells per unit area was estimated and plotted. Phase contrast images of TM cells exposed to the above treatments were captured using a Zeiss Axiovision microscope.

### 2.11 Statistical analysis

Statistical analysis was performed using GraphPad Prism version 7 for Windows (GraphPad Software, La Jolla, CA, USA). All cell biology data represent the average values (mean ± standard error of mean) from at least four independent experiments unless otherwise mentioned. The Mann–Whitney *U*‐test was employed for comparing vasorin levels in human AH samples from glaucomatous and non‐glaucomatous groups. Student's *t*‐test and one‐way analysis of variance (ANOVA) were used for statistical comparisons between any two groups and three groups respectively. A *p* value of >0.05 was considered statistically significant.

## 3 RESULTS

### 3.1 Vasorin is a common constituent of human AH

**FIGURE 1:**
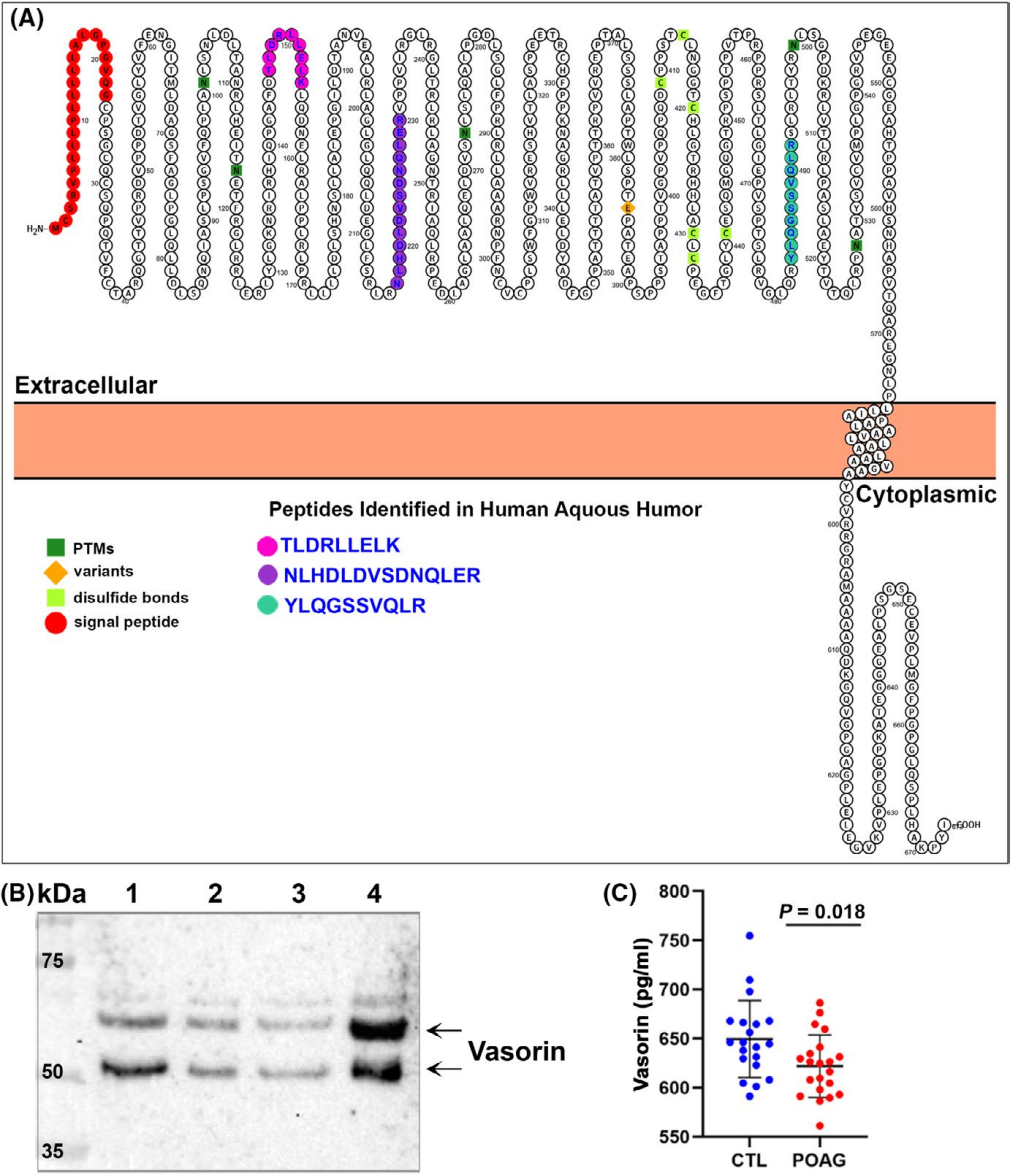
Identification of vasorin in human aqueous humour samples and its decreased levels in POAG patients. (A) Protter tool‐based visualization of vasorin transmembrane topology and the sequence of three individual vasorin peptides derived from the extracellular domain of the protein and detected in human AH samples by mass spectrometry. (B) Detection of vasorin in the AH of non‐glaucoma (cataract) human subjects by immunoblotting analysis. Ten microliters of AH from four individual human donors (lanes 1 to 4) was analysed by immunoblotting analysis. Two prominent immunopositive bands (~65 and 50 kDa, arrows) were identified using monoclonal anti‐vasorin antibody. (C) Vasorin protein levels were significantly but marginally decreased in the AH of POAG patients (*n* = 21) compared to non‐glaucoma (cataract; CTL) patients (*n* = 20) based on the Mann–Whitney *U*‐test. Data represent the median and interquartile range. Individual dots represent results for individual patients/subjects

To identify secretory proteins including vasorin that play a potential role in regulating TM cell biology and AH drainage through the trabecular pathway, we performed mass spectrometry (LC‐MS/MS) based analysis of AH (10 µl) derived from several non‐glaucoma (cataract) patient donors. Vasorin peptides were consistently detected in all AH samples (>10 individual samples) analysed by LC‐MS/MS indicating that vasorin is a common constituent of human AH. Figure 1A shows the three individual peptides identified by LC‐MS/MS, all of which were derived from the extracellular domain of vasorin. Although vasorin was previously identified in human AH,^32^ there have been no studies on the role of vasorin in TM biology and function or in glaucoma. We confirmed the mass spectrometry based identification of vasorin by immunoblotting analysis (Figure 1B) which detected two immunopositive bands corresponding to vasorin species with molecular mass of ~65 and 50 kDa in four representative individual human AH samples.

### 3.2 Decreased levels of vasorin in the AH of POAG patients

Since vasorin is known to be involved in several diseases,^19^ we determined the levels of vasorin in AH derived from twenty‐one POAG patients and twenty age‐ and gender‐matched non‐glaucoma (cataract) patients by ELISA. Table S2 summarizes the demographic details of glaucoma and non‐glaucoma (cataract) patients from whom the AH was collected. In the non‐glaucoma patient group, there were nineteen Caucasians and one Asian, while the glaucoma group consisted of 11 Caucasians and 10 African Americans; therefore, there are significant racial distribution differences between the glaucoma and non‐glaucoma groups. Vasorin levels in the AH of POAG patients (mean ± SEM; 621.89 ± 6.91 pg/ml) were marginally (by 4.4%) but significantly (*p* > 0.05) lower than those in non‐glaucoma patients (649. 58 ± 8.79 pg/ml) based on the Mann–Whitney *U*‐test (Figure 1C). No correlation was found between patients AH vasorin levels and IOP in the POAG group (Figure S1A). Vasorin levels in aqueous humour of POAG and non‐glaucoma patients were also not significantly correlated with patient age (Figure S1B).

### 3.3 Human TM cells express and secrete vasorin

**FIGURE 2:**
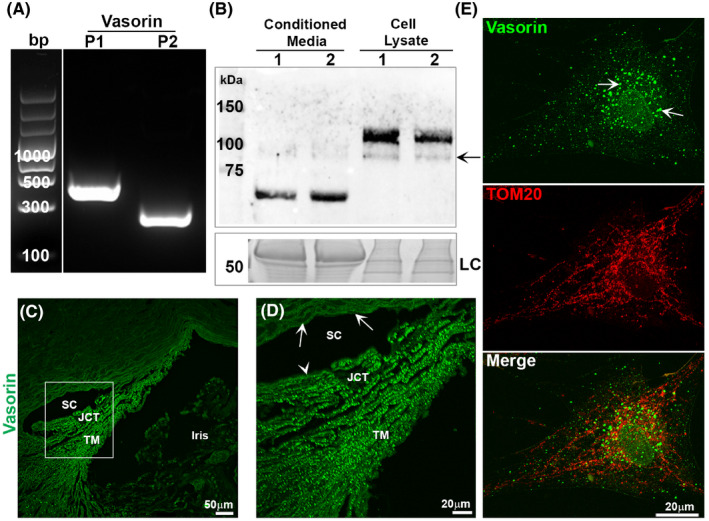
Expression and distribution of vasorin in human trabecular meshwork cells and tissue. (A) RT‐PCR based detection of vasorin expression in HTM cells using two different sets (P1 and P2) of PCR primers. The bp ladder shown in figure was spliced from the same gel in which both the PCR and bp ladder samples were separated and moved from the right side to left side, and a line was placed between the bp ladder lane and PCR products. (B) Detection of vasorin protein by immunoblotting analysis of cell lysates (10 µg protein) and conditioned media (CM; 10 µg protein) derived from two different HTM cell strains (lanes 1 and 2). As expected, the molecular mass of secretory vasorin is smaller than that of the native protein detected in cell lysates, which show a prominent immunopositive band at around ~110 kDa and a second but much weaker immunopositive band migrating below 100 kDa (indicated with an arrow). LC: For loading control, cell lysates and CM samples (10 µg protein per sample) were separated by SDS‐PAGE and stained with GelCode blue stain, a portion of the protein‐stained gel is shown. (C and D) Detection and distribution of vasorin in the HTM by immunofluorescence analysis. Paraffin‐embedded sections derived from the whole globe of the human eye (90 year old) were used for this analysis, and the boxed area in panel C is magnified to show the distribution of vasorin in the trabecular pathway (D). Vasorin exhibits punctate staining (bright green) throughout the trabecular meshwork (TM) including the juxtacanalicular tissue (JCT) with much less to no signal associated with the inner (arrowhead) and outer (arrows) wall of Schlemm's canal (SC) respectively. (E) Distribution of vasorin and TOM20 (mitochondrial protein) in HTM cells. Vasorin reveals a punctate and vesicular (arrows) distribution and does not colocalize with TOM20. Vasorin also shows weaker but positive staining in the nucleus of TM cells (arrowhead). Bars indicate image magnification

Vasorin has been demonstrated to be abundantly expressed in vascular smooth muscle cells.^18^ Since TM tissue exhibits smooth muscle‐like properties and regulates AH outflow and IOP,^33^ we investigated the expression and secretion of vasorin by HTM cells. RT‐PCR analysis confirmed the expression of vasorin, based on the results derived from two different TM cell strains and using two different sets of PCR primers. Figure 2A shows vasorin‐specific PCR products (confirmed by DNA sequencing) in one of the representative TM cell strains derived from a 20‐year‐old donor. Immunoblotting analysis of cell lysates and CM derived from two different HTM lines also confirmed the expression and secretion of vasorin. As expected, vasorin in TM cell lysates (~110 kDa) and CM (~65 kDa) exhibited differences in molecular mass, reflecting the native and cleaved/secreted forms, respectively, of the protein (Figure 2B). In addition to the prominent vasorin immunopositive band above 100 kDa, cell lysates contained a second but weak band below 100 kDa (Figure 2B, arrow). Vasorin was also readily detected in human TM tissue based on immunofluorescence analysis (Figure 2C). The higher magnification image (Figure 2D) reveals a punctate cell surface distribution over the entire TM, with relatively much lesser signal associated with the inner wall of Schlemm's canal (SC), and being almost undetectable in the outer wall of the SC (Figure 2C, D, a representative image from two human specimens is shown). Figure S2 shows vasorin antibody‐specific immunofluorescence in the human trabecular pathway compared to nonspecific fluorescence derived from the use of secondary antibody alone. In HTM cells, vasorin exhibits a vesicular and punctate distribution, with a relatively prominent distribution around the nucleus (Figure 2E, arrows) and marginal distribution in the nucleus (arrowhead). Vasorin does not appear to colocalize with TOM20, a known mitochondrial specific protein, in TM cells (Figure 2E, red).

### 3.4 Regulation of the secreted form of vasorin in TM cells

**FIGURE 3:**
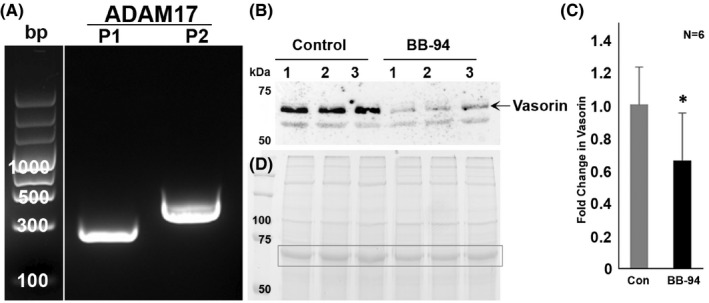
Inhibition of matrix metalloproteinases decreases shedding of the extracellular vasorin domain from trabecular meshwork cells. (A) Confirmation of expression of ADAM17 in human TM cells by RT‐PCR analysis using two sets (P1 & P2) of PCR primers. The bp ladder shown in figure was spliced from the same gel in which both the PCR and bp ladder samples were separated and moved from the right side to left side, and a line was placed between the bp ladder lane and PCR products. (B and C) To understand regulation of secretion of the extracellular form of vasorin (soluble/secretory form) from TM cells, serum starved HTM cells were treated for 24 h with BB‐94 (10 µM), a broad‐spectrum inhibitor of matrix metalloproteinases. CM samples (10 µg protein) from BB‐94 treated TM cells and untreated controls were analysed for the levels of vasorin by immunoblotting analysis. Secreted vasorin (indicated with an arrow) levels were significantly decreased in samples derived from the BB‐94 treated cells compared to control cells, indicating the involvement of matrix metalloproteinases including ADAM17 in shedding of the soluble or active form of vasorin from the TM cells. Lanes 1 to 3 represent three TM cell replicates of the same strain. The second immunopositive band below the prominent band (arrow) did not show much difference between the BB‐94 treated and untreated samples. (D) For loading control, equal amounts of protein from CM samples were separated by SDS‐PAGE and stained with GelCode Blue, an image of the protein‐stained gel is shown and the boxed band was used for normalization to estimate fold change in vasorin protein levels (Panel C). *p < 0.05, *n* = 6

To gain insight into the mechanisms regulating production of the secreted/soluble form of vasorin in HTM cells, we evaluated the expression of ADAM17, which is known to cleave the extracellular part of vasorin to generate the secreted form of the protein.^25^ We confirmed the expression of ADAM17 in two different HTM cell strains by RT‐PCR analysis using two sets of PCR primers (Figure 3A).

Human TM cells maintained under serum‐free conditions (for 24 h) were then treated with a broad‐spectrum matrix metalloproteinase inhibitor, BB‐94 (10 µM) and CM collected for evaluation of soluble vasorin levels by immunoblotting analysis. Treatment of TM cells with BB‐94 resulted in a significant decrease in the levels of the secreted form of vasorin (indicated with arrow) compared to untreated control cells (Figure 3B,C), but did not appear to impact the second, weakly immunopositive lower band present in CM derived from control or inhibitor treated HTM cells (Figure 3B). Figure 3D shows the GelCode blue stained protein profile of CM derived from both control and BB‐94 treated TM cells, with the boxed protein band used as a loading control.

### 3.5 Vasorin induces actin cytoskeletal reorganization and cell adhesive interactions in TM cells

**FIGURE 4:**
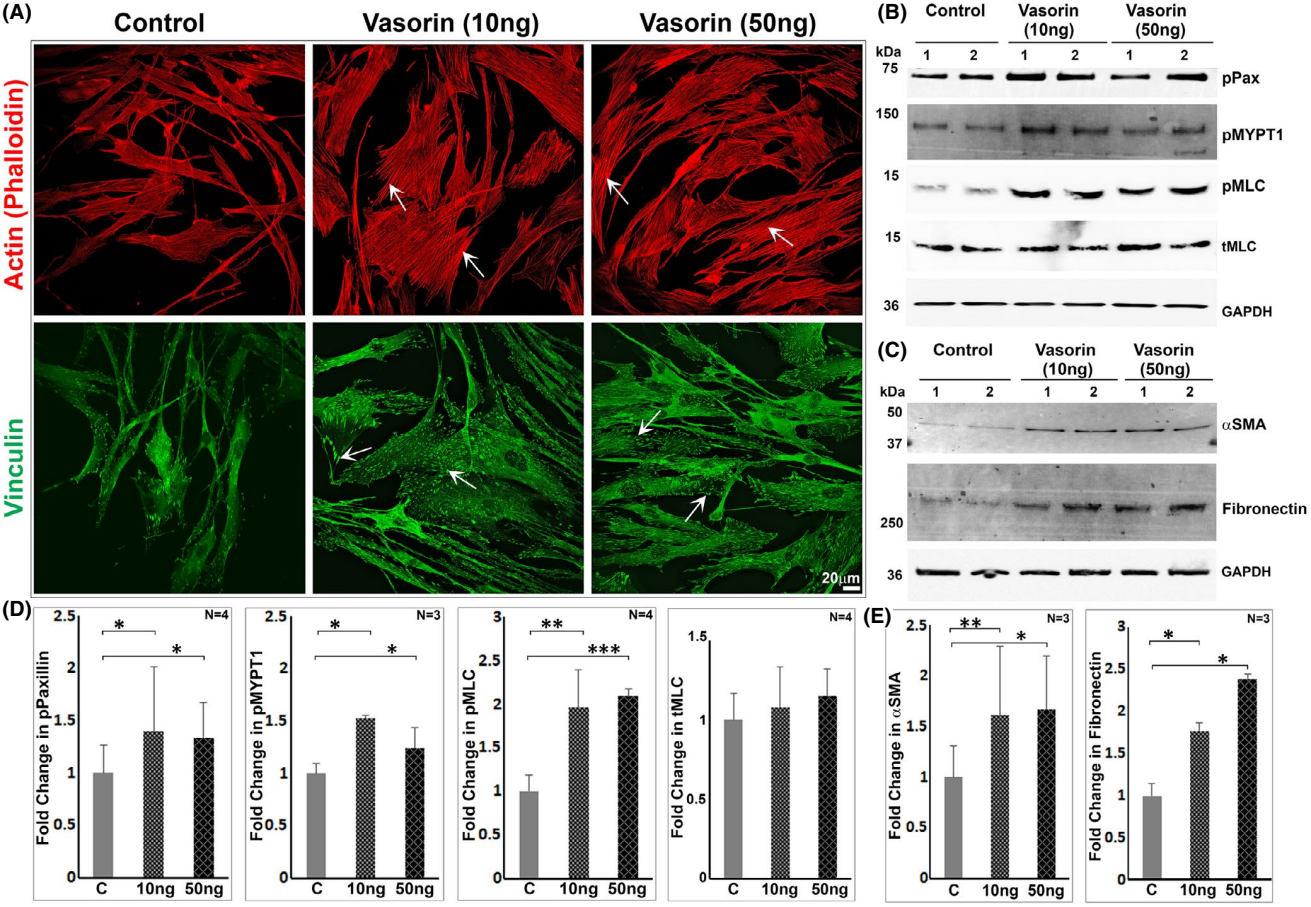
Vasorin induces formation of actin stress fibres and focal adhesions, and the contractile phenotype in trabecular meshwork cells. (A) Treatment of HTM cells maintained under serum‐free conditions (for 24 h) with either 10 or 50 ng vasorin/ml for 24 h led to induction of actin stress fibres (rhodamine‐phalloidin staining, arrows) and focal adhesions (based on vinculin staining, arrows), with cells exhibiting a relatively brisk/stiffer morphology compared to control cells. Images are representative of four biological replicates. Scale bar indicates magnification. (B–E) Consistent with above described conditions and findings, a significant increase was observed in the levels of phospho‐paxillin, phospho‐MYPT1, phospho‐MLC, αSMA and fibronectin in vasorin treated TM cells compared to control TM cells. Total MLC levels were found to be the same between vasorin treated and control samples (B, D) For the estimation of fold change in the levels of p‐Pax, pMYPT1, αSMA and fibronectin, GAPDH blotting was used for normalization. Changes in pMLC levels were normalized to total MLC levels. *p < 0.05; **p < 0.001; ***p < 0.005. In panels D&E, C = control

The extracellular region of vasorin contains several motifs including tandemly organized leucine‐rich repeat regions, an epidermal growth factor ‐like repeat and a fibronectin type III domain, indicating the plausible involvement of vasorin in cell adhesive interactions and cytoskeletal reorganization via interaction with cell surface proteins. To explore this premise, HTM cells maintained under serum‐free conditions for 24 h were treated with soluble vasorin (10 and 50 ng/ml) for 24 h. Cells treated with vasorin exhibited induction of actin stress fibre formation, (Phalloidin staining, red), focal adhesions (vinculin distribution, green) and changes in contractility (visual assessment of contractile/stiffer morphology), relative to untreated controls (Figure 4A). Vasorin treatment also led to a significant increase in the levels of phospho‐paxillin (p‐Pax), phospho‐MYPT1 (pMYPT1; one of the targets of Rho kinase), phospho‐myosin light chain (pMLC, the regulatory subunit of myosin II), ^34^ α‐SMA and fibronectin in HTM cells (Figure 4B‐E). Total MLC levels were found to be not different between the vasorin treated and control TM cells (Figure 4B,D). Figure 4D depicts the fold change in levels of p‐Pax and pMYPT1 under vasorin treatment, with GAPDH used as loading control. Total MLC and GAPDH were used as loading controls, respectively, to calculate fold changes in pMLC and total MLC levels (Figure 4D). Collectively, these results argue a role for soluble vasorin in influencing the contractile and cell adhesive characteristics of TM cells.

### 3.6 Vasorin‐mediated mitigation of TGF‐β2‐induced changes in TM cells

**FIGURE 5:**
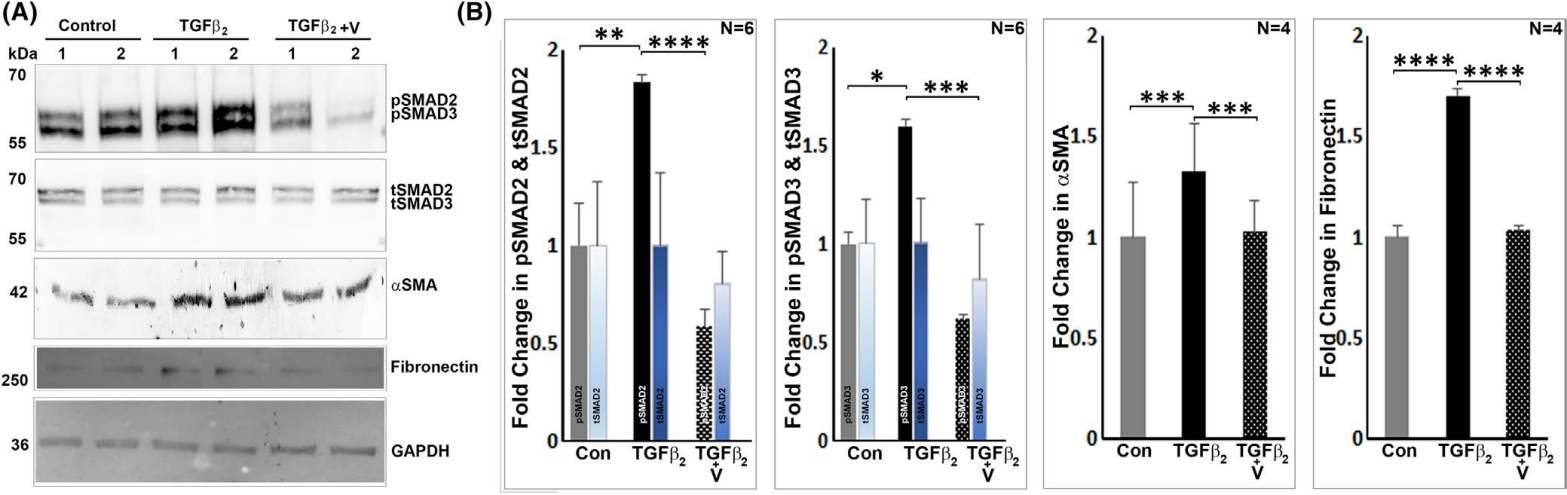
Vasorin suppresses TGF‐β2 induced responses in trabecular meshwork cells. (A, B) Human TM cells maintained under serum‐free conditions (24 h) were treated with TGF‐β2 (2 ng/ml) for 24 h in the absence or presence of vasorin (50 ng/ml), prior to analysis of TGF‐ β2 induced activation of SMAD2 and SMAD3, and upregulation of αSMA and fibronectin expression. Under these conditions, while TGF‐β2 significantly increased the levels of phospho‐SMAD2 (pSMAD2) and phospho‐SMAD3 (pSMAD3), αSMA and fibronectin, these changes were significantly suppressed in the presence of vasorin indicating the inhibition of TGF‐β2 responses by vasorin in TM cells. The levels of total SMAD2 and SMAD3 were comparable between control and TGF‐β2 or TGF‐β2 plus vasorin treated samples (A, B) Fold changes shown in the levels of fibronectin, αSMA and total SMAD2 and SMAD3 were based on GAPDH loading control. Total SMAD2 and SMAD3 levels were used to normalize the levels of pSMAD2 and pSMAD3 respectively. **p < 0.001; ***p < 0.005; ****p < 0.0001. V: Vasorin, Con: Control, tSMAD2: total SMAD2, tSMAD3: total SMAD3, pSMAD2: phospho‐SMAD2, pSMAD3: phospho‐SMAD3

One of the well‐recognized biological activities of vasorin is trapping and blocking of TGF‐β activity,^18^, ^25^ and suppressing the TGF‐β‐induced epithelial‐to‐mesenchymal transition (EMT).^25^ Since elevated levels of TGF‐β, EndMT (endothelial‐to‐mesenchymal transition) and fibrogenic activities are presumed to play a part in ocular hypertension and glaucoma,^33^, ^35^, ^36^ we determined the effects of vasorin on TGF‐β2 mediated activation of SMAD2/3, and expression of α‐SMA and fibronectin in TM cells. While treatment of serum starved (24 hrs) TM cells with TGF‐β2 (2 ng/ml for 24 h) led to a significant increase in the levels of phospho‐SMAD2 and SMAD3, and stimulated expression of α‐SMA and fibronectin, the presence of vasorin (50 ng/ml for 24 h) together with TGF‐β2 (Figure 5A, B) resulted in significant suppression of these changes. The levels of total SMAD2 and SMAD3 were found to be unchanged in the above described samples (Figure 5A, B). These results imply the ability of vasorin to block TGF‐β induced changes in TM cells.

### 3.7 Hypoxia upregulates vasorin expression in human TM cells

**FIGURE 6:**
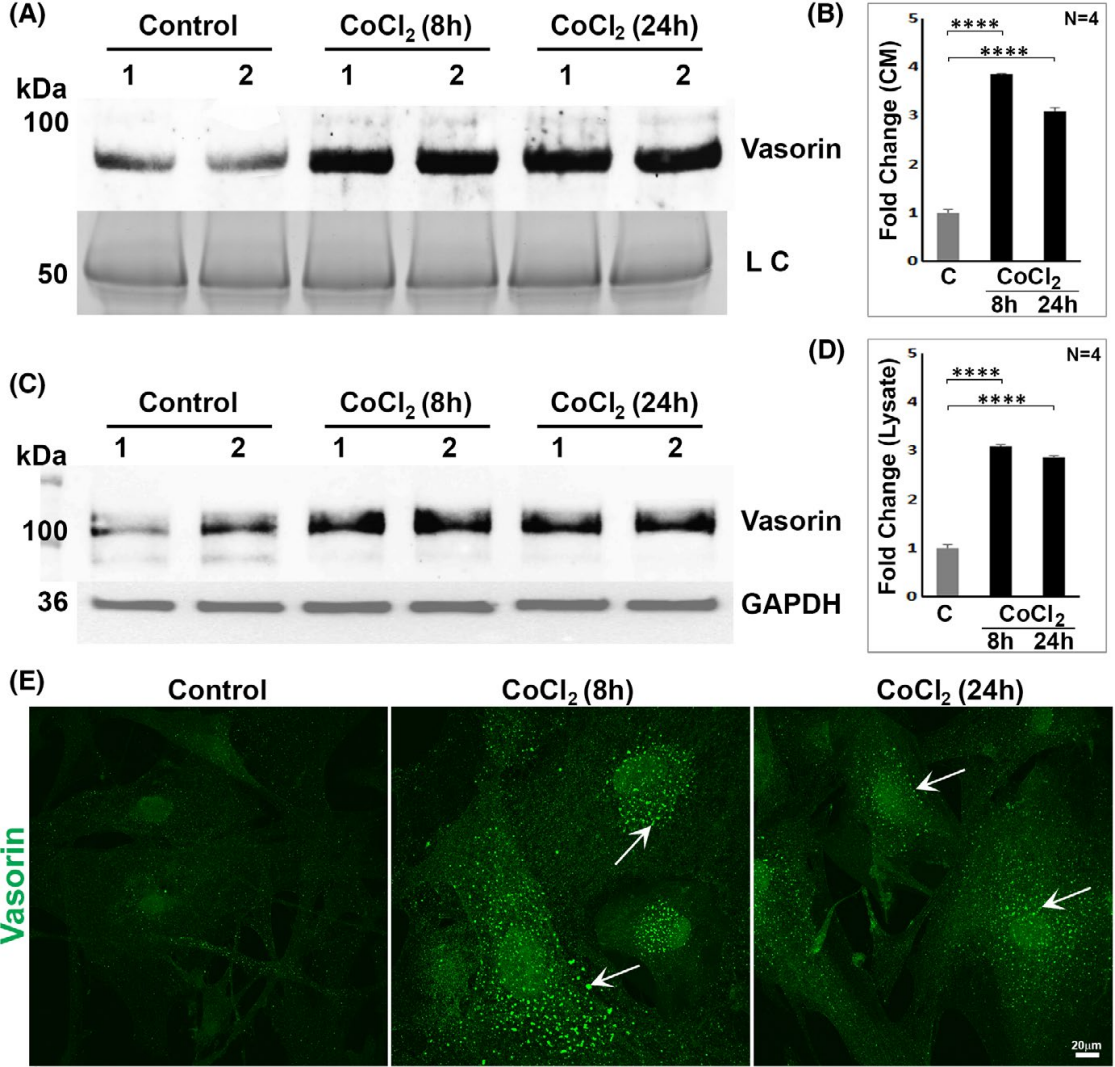
Upregulation of vasorin by cobalt chloride‐induced hypoxia in TM cells. To test whether hypoxia induces vasorin expression in TM cells, conditioned media (CM) (5 µg protein; A, B) and cell lysates (10 µg protein; C, D) samples derived from HTM cells maintained under serum‐free condition (for 24 hrs) prior to treatment with CoCl_2_ (0.4 mM) for 8 and 24 h were immunoblotted for vasorin. CoCl_2_ treatment significantly elevated vasorin levels in both CM and lysates of TM cells treated for both 8 and 24 h compared to control cells. For the CM samples, proteins separated by SDS‐PAGE gel were stained with GelCode blue and one of the indicated protein bands was used as loading control (LC). For cell lysates, GAPDH was used as a loading control. ****p < 0.0001. In panels B & D, C = control: (E). In addition to immunoblotting analysis, TM cells were also immunostained for vasorin. As shown in the figure, cells treated with CoCl_2_ revealed increased immunofluorescence for vasorin (punctate/vesicular distribution pattern, indicated with arrows) compared to untreated control cells. Scale bar indicates image magnification

Vasorin has been reported to possess tumour growth promoting activity in glioblastoma cells, anti‐apoptotic activity, and to exhibit upregulated expression in mouse embryonic fibroblasts under hypoxic conditions.^20^, ^21^ To determine whether hypoxia influences vasorin expression in TM cells, cell lysates and CM derived from HTM cells (serum starved for 24 h) treated with CoCl_2_ (0.4 mM) for 8 and 24 h were subjected to vasorin immunoblotting analysis. Vasorin levels were robustly and significantly increased in both CM (Figure 6A, B) and cell lysates (Figure 6C, D) under CoCl_2_‐induced hypoxic conditions at both the 8‐ and 24‐hr intervals relative to control cells. We also immunostained TM cells for vasorin under similar conditions, and as shown in Figure 6E, observed an increase in vasorin immunostaining that exhibited a vesicular/punctate distribution pattern in CoCl_2_ treated cells relative to untreated controls. These findings confirm that hypoxic conditions upregulate vasorin expression in TM cells.

### 3.8 Vasorin suppresses TNF‐α induced cell death in TM cells

**FIGURE 7:**
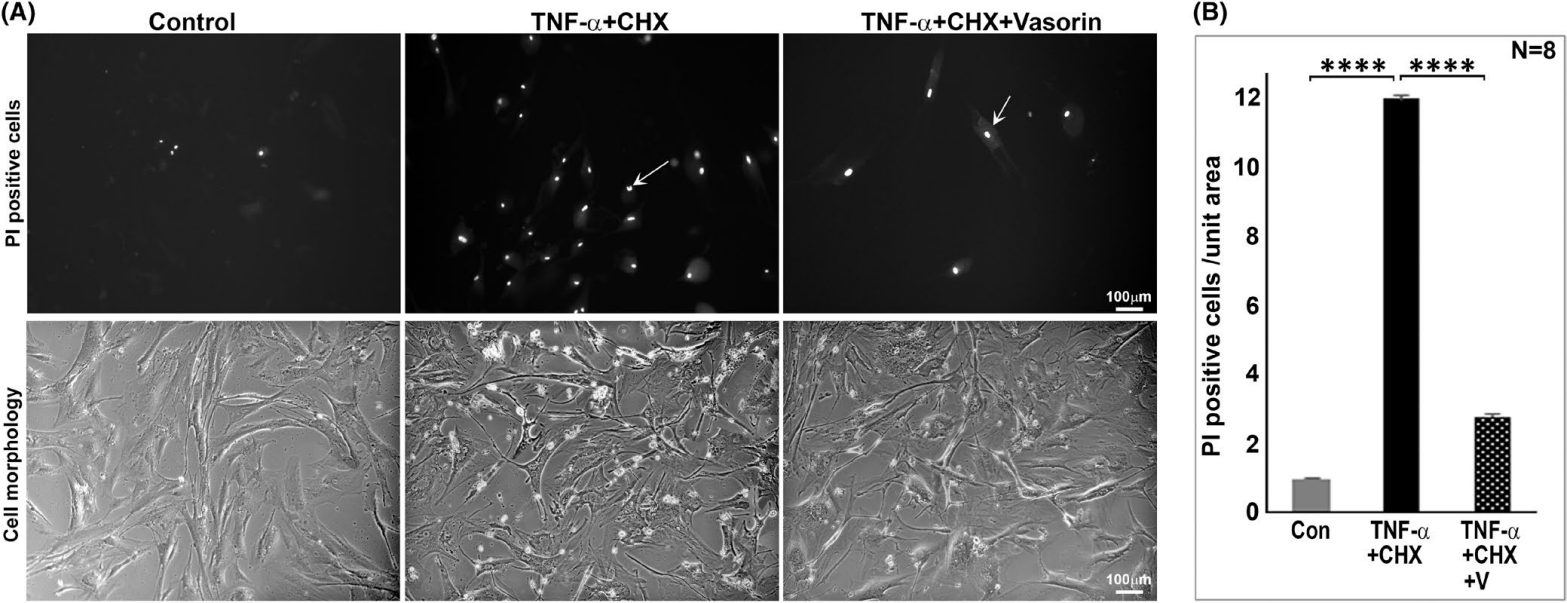
Suppression of TNF‐α induced TM cell death by vasorin. (A, B) To evaluate the effects of vasorin on TNF‐α induced TM cell death, HTM cells maintained under serum‐free condition (for 24 h) were treated with TNF‐α (30 ng/ml) and cycloheximide (CHX, 1 µg/ml) for 24 h in the absence or presence of vasorin (50 ng/ml). Following this treatment, live cells were stained with propidium iodide (PI), imaged and counted for PI positive staining (arrow). TM cells treated with TNF‐α and cycloheximide exhibited a significant increase in cell death (PI positive cells) compared to control cells. The effect of TNF‐α on cell death was significantly reduced in the presence of vasorin (V). (A) The lower panels show phase contrast images of HTM cells under the above described conditions. Relatively, the TNF‐α and cycloheximide treated cells exhibit notable changes in cell morphology with punctate appearance compared to control and vasorin supplemented cells. ****p < 0.0001. Bars indicate image magnification. Con: Control

Having found robust upregulation of vasorin expression under hypoxia, we evaluated the effects of vasorin on the survival of TM cells treated with TNF‐α. AH derived from POAG patients has been shown to have elevated levels of TNF‐α,^10^ and TM tissue derived from glaucoma patients has been documented to exhibit reduced TM cell density.^14^ Moreover, vasorin has been shown to protect mouse embryonic fibroblasts from TNF‐α induced apoptotic cell death.^21^ Confluent cultures of serum starved (24 h) HTM cells treated with TNF‐α (30 ng/ml) and cycloheximide (1 µg/ml) for 24 h revealed a significant increase in cell death relative to untreated controls, as assessed by propidium iodide (PI) labelling (Figure 7A, B). Addition of vasorin to TM cells together with TNF‐α and cyclohexamide resulted in a significant decrease in cell death relative to that observed in TM cells treated with TNF‐α and cyclohexamide (Figure 7A, B), revealing that vasorin protects TM cells from TNF‐α‐induced cell death. The lower panels in Figure 7A show phase contrast images of HTM cell morphology under the above described treatments. Relatively, the TNF‐α and cycloheximide treated cells exhibit notable changes in cell morphology with punctate appearance compared to control and vasorin supplemented cells.

## 4 DISCUSSION

While AH outflow through the trabecular meshwork, and IOP are modulated by a variety of extracellular inputs including physiological growth factors whose dysregulation is associated with ocular hypertensive glaucoma,^7^, ^11^ we are yet to establish a complete understanding regarding identity of the external cues regulating AH drainage and IOP. This study identifies vasorin as a common constituent of AH, demonstrates that expression of this glycoprotein which is expressed and secreted by TM cells, is induced in response to hypoxic conditions, and that vasorin stimulates actin cytoskeletal reorganization, focal adhesion formation and contractile changes in TM cells. Importantly, vasorin appears to block the biological effects of TGF‐β and prevent TNF‐α induced cell death in TM cells. Finally, AH samples from POAG patients exhibited a propensity to contain lower levels of vasorin. Collectively, these experimental and clinical findings indicate not only a role for vasorin in regulating TM cell physiology and survival, but also suggest that vasorin may possess potential as a therapeutic agent for lowering IOP in glaucoma patients.

Though we identified vasorin as a common constituent of human AH and confirmed that this glycoprotein is expressed by TM cells, we have no knowledge regarding the regulation of vasorin expression in and secretion from TM cells and tissue, or how vasorin levels are regulated in the AH. HTM cells and tissue were confirmed to express vasorin, and TM cells demonstrated to secrete vasorin through shedding or proteolytic cleavage of the extracellular region of the protein by matrix metalloproteinases including ADAM17. Although we did not confirm the direct involvement of ADAM17 in production and release of active and soluble vasorin in this study, BB‐94, which is a broad‐spectrum inhibitor of matrix metalloproteinases, was found to inhibit the secretion of soluble vasorin from HTM cells in our study. BB‐94 has been shown to suppress the production of soluble vasorin through inhibition of ADAM17 in MCF7 breast cancer cells.^25^ Therefore, we speculate that ADAM17, which is readily detected in TM cells, participates in regulation of secretion of the active form of vasorin. While the predominant species of intracellular vasorin in TM cells exhibits an expected molecular mass of 110 kDa, we also detected a weaker immunoreactive band migrating slightly lower than 100 kDa in some of the immunoblots. Vasorin is not only glycosylated but has also been reported to be sialylated;^37^ it is therefore possible that the weaker band migrating lower than 100 kDa represents a differentially post‐translationally modified form of vasorin. Similarly, the secreted forms of vasorin found in the CM of TM cells and in the AH were in the range of 70–50 kDa, and these molecular masses are lower than the expected and reported molecular mass of ~90 kDa.^25^ The reason for this discrepancy is not clear, and the question of whether the secreted form of vasorin is a target of matrix metalloproteinase type II and other proteases in the AH needs to be evaluated in future studies.^38^

Vasorin expression was robustly induced under CoCl_2_‐induced hypoxia in TM cells. Vasorin is a HIF‐1α/STAT3 target protein whose expression is induced under hypoxic conditions in different cell types.^20^, ^21^ CoCl_2_ treatment of TM cells also led to increased secretion of vasorin. Although the cellular effects of increased vasorin secretion under hypoxic condition have not been addressed in this study, it is possible that hypoxia‐induced vasorin alters the TM cell contractile and relaxation properties of TM cells which possess certain characteristics of vascular smooth muscle and endothelial cells, and hypoxia is known to regulate vasoconstriction and relaxation of the latter in a tissue‐specific manner.^39^, ^40^ Interestingly, we found that soluble (secretory) vasorin induced actin stress fibres, focal adhesions and myosin light chain phosphorylation in TM cells, indicating that vasorin can influence the contractile properties of TM cells, which are known to influence AH outflow through the TM and IOP.^33^ However, the mechanisms utilized by extracellular vasorin to influence cell adhesive interactions and actin cytoskeletal organization are not clear at this time. Whether vasorin mediates these effects via interaction with, yet to be identified cell surface receptors and ion channels that in turn influence intracellular calcium flux, myosin light chain kinase or Rho/Rho kinase activation, needs to be explored in future studies. It is noteworthy that decorin, which exhibits sequence homology with varosin especially in the tandemly organized leucine‐rich repeat regions,^18^ has been shown to induce actin cytoskeletal changes in various cell types including TM cells.^41^, ^42^

The well‐recognized physiological characteristics of vasorin include a role in regulation of TGF‐β activity and inhibition of TNF‐α induced apoptosis.^18^, ^21^ Vasorin has been demonstrated to directly bind TGF‐β and block TGF‐β signalling activity including the fibrotic response and EMT in different cell types.^18^, ^25^, ^38^ Importantly, elevated levels of TGF‐β is not only a common finding in the AH of POAG patients, but increased levels of TGF‐β are recognized to elevate IOP by stimulating fibrogenic activity and transdifferentiation of TM cells into extracellular matrix producing fibroblast‐like cells.^11^, ^27^, ^35^ In this study, vasorin was found to block the biological activity of TGF‐β2 as evidenced by suppression of TGF‐β2–induced SMAD2/3 activation, and expression of α‐SMA and fibronectin in TM cells. Intriguingly, vasorin per se appears to induce contractile activity and expression of αSMA and fibronectin in TM cells. In contrast, vasorin appears to mitigate the TGF‐β induced effects on α‐SMA and fibronectin suggesting that vasorin possesses a higher binding affinity to bind and block the activity of TGF‐β relative to other vasorin binding proteins. Additionally, TGF‐β‐induced fibrogenic activity has been shown to be augmented in the absence of vasorin, indicating the importance of varosin expression in regulation of TGF‐β activity.^18^, ^25^, ^38^

Vasorin has also been demonstrated to suppress hypoxia‐ and TNF‐α‐induced apoptosis partly through regulation of thioredoxin anti‐oxidative activity and generation of reactive oxygen species by mitochondria.^21^ Since elevated levels of TNF‐α in the AH and loss of TM cells are common characteristics of glaucoma,^10^, ^14^ and hypoxia was observed to robustly upregulate expression of vasorin in TM cells, we evaluated the effects of vasorin on TNF‐α induced cell death and found that vasorin suppresses TNF‐α induced cell death in TM cells. The mechanism underlying this effect of vasorin, however, is not as clear as has been documented in mouse embryonic fibroblasts where the anti‐apoptotic activity of vasorin against TNF‐α has been shown to be mediated through thioredoxin activity in mitochondria.^21^ In TM cells, we did not detect vasorin distributing to mitochondria, with the protein exhibiting vesicular and some nuclear localization. In this study, we did not determine the levels of Notch1 or Numb in TM cells. Elevated levels of vasorin have been demonstrated to bind to and prevent the degradation of Notch1 in glioblastoma, and the resulting augmentation of Notch1 stability is thought to account for the vasorin‐associated aggressiveness of glioma.^20^

Finally, our assessment of whether dysregulation of vasorin, especially the secreted form of vasorin could be linked to POAG which is commonly associated with ocular hypertension,^5^ revealed only a trend towards significantly lowered levels of vasorin in AH derived from POAG patients compared to age‐ and gender‐matched non‐glaucoma patients. Although the decrease in vasorin levels was marginal, this decreasing trend suggests the potential for augmentation of TGF‐β‐induced fibrogenic activity in TM cells and TM cell transdifferentiation. Additionally, vasorin expression is known to be decreased with ageing and by angiotensin II, leading to augmentation of TGF‐β induced fibrotic activity in the vascular smooth muscle cells of arterial wall,^38^ and the role of angiotensin II in ocular hypertension and glaucoma is well‐recognized.^43^ Taken together, this study sheds light on novel findings regarding the role of vasorin in TM cells and the propensity for decreased vasorin levels in the AH of glaucoma patients. Vasorin was found to exhibit anti‐TGF‐β and anti‐apoptotic activity in TM cells implying that dysregulation of production and/or secretion of this key glycoprotein by TM cells may be contributory to the pathobiology of ocular hypertension and glaucoma and that vasorin may therefore be a therapeutic target in glaucoma.

## CONFLICTS OF INTEREST

The authors have no financial and/or non‐financial interests in relation to the work described.

## AUTHOR CONTRIBUTION

**Jin A. Choi:** Conceptualization (equal); Formal analysis (equal); Investigation (equal); Writing – original draft (equal). **Rupalatha Maddala:** Conceptualization (equal); Formal analysis (equal); Investigation (equal); Validation (equal); Writing – original draft (equal). **Shruthi Karnam:** Formal analysis (equal); Resources (equal); Writing – original draft (supporting). **Nikolai P. Skiba:** Formal analysis (equal); Methodology (equal); Writing – original draft (supporting). **Robin Vann:** Conceptualization (equal); Resources (equal). **Pratap Challa:** Conceptualization (equal); Resources (equal); Writing – original draft (supporting). **Ponugoti Vasantha Rao:** Conceptualization (lead); Funding acquisition (lead); Project administration (lead); Writing – review & editing (lead).

## Supporting information

Supplementary Material

## Data Availability

Data available on request from the authors.
