# Supplementary material for: Role of vasorin, an anti‐apoptotic, anti‐TGF‐β and hypoxia‐induced glycoprotein in the trabecular meshwork cells and glaucoma
Source: J Cell Mol Med. 2022 Feb 16;26(7):2063–75. doi: 10.1111/jcmm.17229 (PMC8980963; doi:10.1111/jcmm.17229)
Supplement: Supplementary file 1 — Supplementary Material [file JCMM-26-2063-s001.docx]

**Figure. S1A.**

**Fig. S1A. Vasorin levels in the aqueous humor of POAG patients did not show a significant correlation with IOP.** No correlation was found between patient AH vasorin levels and IOP in the POAG group (Pearson Correlation Coefficient = -0.027, 95% CI = -0.4879 to 0.4453, p = 0.9146). Data represent the mean linear regression (black line) and the 95% confidence interval (black dotted line).

**Figure. S1B.**

**Fig. S1B. Varosin levels in aqueous humor of POAG and non-glaucoma patients did not show a significant correlation with patient’s age.** No correlation was found between patient age and AH vasorin levels of both POAG and non-glaucoma group (Pearson Correlation Coefficient = 0.099, 95% CI = -0.273 to 0.444, p = 0.538). Data represent the mean linear regression (black line) and the 95% confidence interval (black dotted line).

**Figure. S2.**


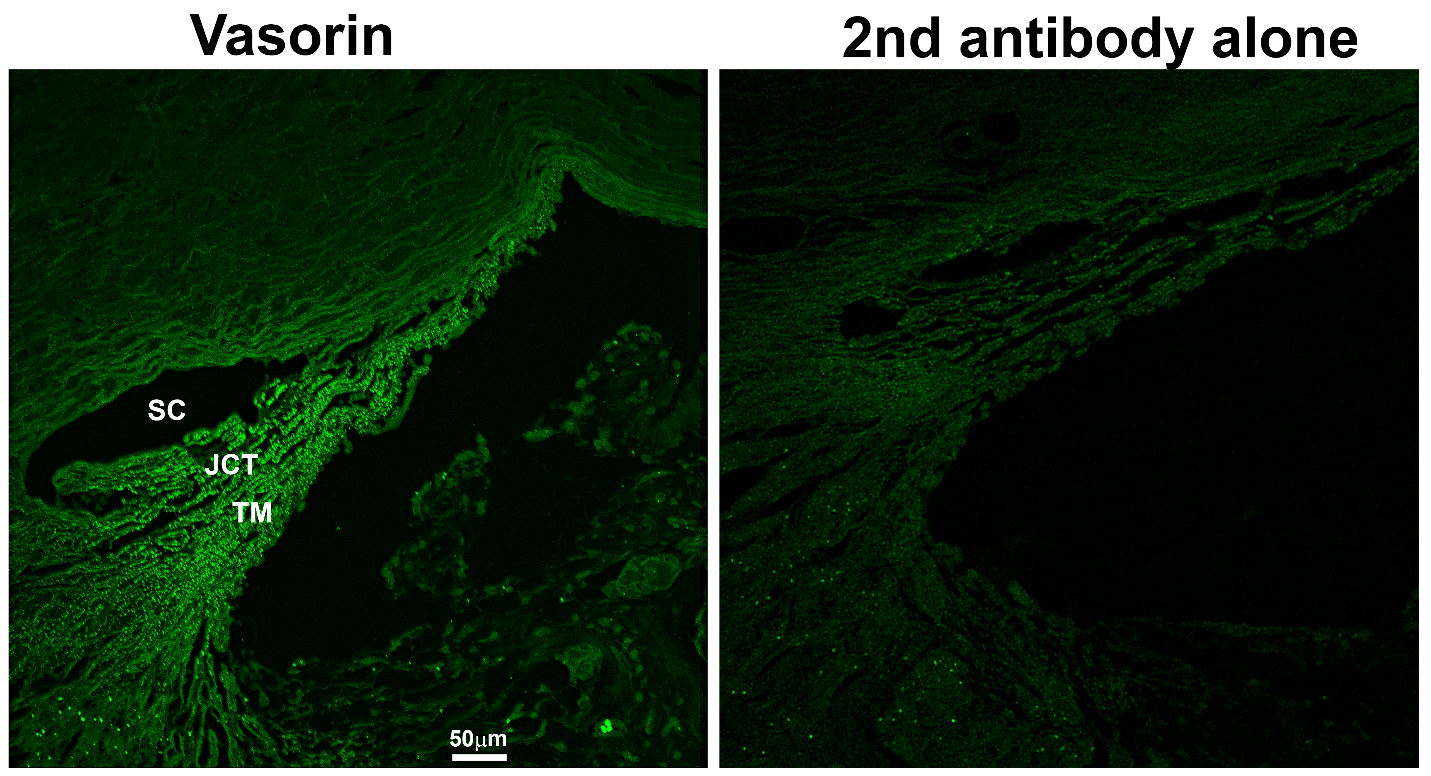


**Fig. S2**. **Specificity of vasorin antibody used in the immunofluorescence-based detection of vasorin distribution in the trabecular meshwork pathway of human donor eye.** Compared to specimens treated with secondary antibody alone (Alexa Fluor 488), these is an intense and specific immunofluorescence (green) staining distributing in the trabecular meshwork in the specimen treated with both vasorin primary antibody and secondary antibody, confirming the specificity of primary antibody used.

**Table S1**. Details of antibodies used for immunoblotting (IB) and immunofluorescence (IF) analyses:

| **Antibodies** | **Cat. No.** | **Source** | **Dilution (IB)** | **Dilution (IF)** |
| --- | --- | --- | --- | --- |
| Human Vasorin/SLIT‑like 2 mouse monoclonal | MAB2140 | R&D Systems, Minneapolis, MN | 1:500 | 1:200 |
| Tom20 rabbit polyclonal | SC-11415 | Santa Cruz Biotechnology, Inc. Dallas, Texas |  | 1:1000 |
| Phalloidin–Tetramethyl rhodamine B isothiocyanate (TRITC) | P1951 | Sigma/Aldrich, St. Louis, MO |  | 1:500 |
| Vinculin mouse monoclonal | V9131 | Sigma/Aldrich, St. Louis, MO |  | 1:250 |
| GAPDH mouse monoclonal | 60004-1 | Proteintech Group, Chicago, IL | 1:8000 |  |
| Propidium Iodide | P4170 | Sigma/Aldrich, St. Louis, MO |  | 1:2000 |
| Hoechst 33258, penta hydrate (bis-benzimide) | H21491 | Thermo Fisher Scientific.  Eugene, OR |  | 1:5000 |
| Phospho-Paxillin (Tyr118) Rabbit polyclonal | 2541 | Cell Signaling Technology, Danvers, MA | 1:1000 |  |
| Phospho-MYPT1 (Thr696) Rabbit polyclonal | ABS45 | Millipore, Billerica, MA | 1:1000 |  |
| Phospho-Myosin Light Chain 2 (Thr18/Ser19) Rabbit polyclonal | 3674 | Cell Signaling Technology, Danvers, MA | 1:1000 |  |
| Myosin Light Chain Rabbit polyclonal | 3672S | Cell Signaling Technology, Danvers, MA | 1:1000 |  |
| αSMA clone 1A4 mouse monoclonal | A2547 | Sigma-Aldrich, St. Louis, MO | 1:2000 |  |
| Fibronectin rabbit polyclonal |  | Harold P Erickson, Duke University | 1:8000 |  |
| Phospho-Smad2 (Ser465/ 467)/Smad3 (Ser423/425) rabbit monoclonal | 8828 | Cell Signaling Technology, Danvers, MA | 1:1000 |  |
| Smad2/3 Antibody rabbit polyclonal | 3102S | Cell Signaling Technology, Danvers, MA | 1:1000 |  |

Secondary antibodies for immunofluorescence analyses:

| **Antibodies** | **Cat. No.** | **Source** | **Dilution** |
| --- | --- | --- | --- |
| Alexa Fluor^TM^ 488 goat anti-Rabbit IgG | A11077 | Invitrogen / Thermo Fisher Scientific. Rockford, IL | 1:500 |
| Alexa Fluor^TM^ 568 goat anti-mouse IgG | A11004 | Invitrogen / Thermo Fisher Scientific. Rockford, IL | 1:200 |

Secondary antibodies for immunoblot analyses:

| **Antibodies** | **Cat. No.** | **Source** | **Dilution** |
| --- | --- | --- | --- |
| Peroxidase AffiniPure Goat Anti-Rabbit IgG (H+L) | 111-035-144 | Jackson ImmunoResearch Inc, West Grove, PA | 1:5000 |
| Goat anti-Mouse IgG (H+L) Secondary Antibody, HRP | 31430 | Invitrogen / Thermo Fisher Scientific. Rockford, IL | 1:5000 |

**Table S2: Demographics of POAG and non-glaucoma (cataract) subject groups**.

|  | **Non-glaucoma (cataract)** | **POAG** | ***P* Value** |
| --- | --- | --- | --- |
| **Number** | **20** | **21** |  |
| **Age (years)** | **72.7 ± 7.3** | **69.6 ± 10.8** | **0.216*** |
| **Sex** |  |  |  |
| **Male** | **8** | **8** | **1.000^†^** |
| **Female** | **12** | **13** |  |
| **Race** |  |  |  |
| **Caucasian** | **19** | **11** | **0.001^†^** |
| **African-American** | **0** | **10** |  |
| **Asian** | **1** | **0** |  |

***Mann-Whitney U-test, †Chi-square test**
